# Testbed Design and Performance Emulation for Satellite–Terrestrial Integrated Networks

**DOI:** 10.3390/s26144623

**Published:** 2026-07-21

**Authors:** Erlong Wei, Junna Yu, Yihong Wen

**Affiliations:** 1 School of Artificial Intelligence, Xidian University, Xi’an 710071, China; weiel@stu.xidian.edu.cn; 2 The 54th Research Institute of China Electronics Technology Group Corporation, Shijiazhuang 050081, China; kungfu_w@126.com

**Keywords:** satellite–terrestrial integrated networks, non-terrestrial networks, software testbed, performance emulation, sensor networks, remote IoT, resource scheduling

## Abstract

Satellite–terrestrial integrated networks (STINs) can extend remote sensor telemetry, remote Internet of Things (IoT), and emergency communication services beyond terrestrial coverage, but their evaluation is complicated by heterogeneous mobility, channel, resource, and control-plane dynamics. This study presents a software-based modular testbed and performance-emulation framework for STINs. The framework integrates scenario generation, model-driven data processing, replaceable algorithm engines, scheduler-based execution control, and a Kafka-style message interface. It models terrestrial, unmanned aerial vehicle, and low-Earth-orbit satellite entities and provides link-budget abstraction, access control, mobility-aware handover, traffic generation, scheduling, load balancing, adaptive routing, and multi-mode transmission for mixed sensing and communication traffic. The representative strategies are evaluated using a lightweight emulation model parameterized by standards-informed NTN and link-budget assumptions. Representative results reveal tradeoffs between access, handover, routing, and scheduling strategies, together with sensitivity to workload, mobility, outage, demand, and selected model parameters. The proposed framework therefore supports traceable STIN strategy evaluation for remote sensor networks, sensing-data backhaul, and remote-IoT service scenarios under explicit emulation assumptions.

## 1. Introduction

Future wireless systems are expected to provide connectivity across terrestrial, aerial, maritime, and remote-area environments [1]. Industrial Internet of Things (IoT), smart-grid monitoring, environmental sensing, smart agriculture, and emergency response all require persistent connectivity beyond densely deployed terrestrial networks. For example, in industrial IoT, outdoor logistics assets, agricultural automation devices, offshore equipment, and fishery monitoring terminals need reliable telemetry and control when terrestrial coverage is unavailable or disrupted [2]. Smart-grid applications similarly rely on wide-area monitoring, fault recovery, distributed-energy coordination, and emergency restoration under infrastructure failures [3]. Although 4G and 5G networks improve terrestrial access, sparse deployment, massive heterogeneous demand, and infrastructure disruption still limit coverage, resource coordination, and service resilience in these scenarios [4,5,6,7].

Satellite–terrestrial integrated networks (STINs) provide a promising approach to mitigating these limitations by combining terrestrial cellular infrastructure with satellite coverage. Low-Earth-orbit (LEO) constellations are especially relevant because they offer lower propagation delay and finer coverage granularity than traditional high-orbit satellite systems [8,9]. By integrating LEO satellite links, terrestrial cellular links, gateways, and edge or core network functions, STINs can support coverage extension, cross-domain traffic steering, and resilient service access for remote sensing, emergency communication, sensor-data collection, and IoT applications [10,11]. The standardization of non-terrestrial networks (NTNs) in 3GPP has further clarified deployment scenarios, channel assumptions, architectural impacts, and solution directions for integrating satellite access with New Radio (NR) systems [12,13,14].

Despite these advances, platform-level evaluation of STIN strategies remains difficult. Satellite visibility, beam-level resources, propagation delay, Doppler-related dynamics, terrestrial cell load, user mobility, traffic diversity, and backhaul reachability interact across different time scales [15]. Access selection decisions may reshape load and handover behavior, routing decisions may affect reachability and delay, and scheduling determines whether heterogeneous services meet their rate and latency targets [16,17]. Consequently, evaluating one function in isolation can hide cross-module tradeoffs. A useful early-stage environment should therefore run replaceable control algorithms on common terrestrial, UAV, and satellite states and export traceable metrics under identical traffic, visibility, outage, and priority settings. This requirement motivates a lightweight platform-level workflow between packet-level protocol simulation and deployment-calibrated digital twins.

### 1.1. Related Work

STIN evaluation cannot be reduced to a single channel model, access rule, or resource-allocation problem. A software testbed must first describe terrestrial, aerial, and satellite entities in a common scenario and then preserve the link, mobility, traffic, and load differences that affect network decisions. Architecture, routing, and resource-management studies show why these aspects are coupled, because STIN performance depends jointly on mobility, satellite visibility, spectrum use, backhaul reachability, traffic demand, and service priority [18,19,20,21,22]. Channel- and link-level studies further indicate that terrestrial and satellite candidates should be evaluated through consistent propagation, link-budget, and physical-layer abstractions before access or routing decisions are compared [23,24,25].

Existing evaluation tools provide useful but incomplete support for this objective. General network simulators such as ns-3 and OPNET are suitable when packet-level or protocol-level fidelity is the main goal, and recent extensions have improved NTN-related channel modeling [26,27]. Satellite-network simulators are effective for constellation visibility, topology evolution, and satellite-link analysis [28,29]. Digital-twin [30,31] and modular-testbed [32] studies move closer to integrated closed-loop validation, especially when calibrated models, deployment interfaces, or experimental infrastructure are available. Technology-specific experimental platforms provide a complementary validation path. For example, the LoRaWAN satellite-backhaul testbed in [33] focuses on the design and validation of a concrete IoT backhaul configuration. Such platforms provide stronger implementation evidence for their target technology, whereas the present work provides a broader software-level workflow for comparing replaceable STIN control functions. However, early-stage strategy evaluation also needs a compact software workflow in which multiple algorithms can process the same scenario records, use the same timing assumptions, and export comparable metrics. The proposed workflow therefore complements, rather than replaces, packet-level simulators, technology-specific testbeds, and deployment-calibrated digital twins. This work is therefore positioned between packet-level protocol simulation and deployment-calibrated digital twins; it focuses on software-level integration, repeatable record exchange, replaceable decision engines, and comparable metric export for STIN control strategies.

Algorithm-oriented studies provide important candidate engines for such a workflow. Existing work has examined satellite resource scheduling, beam control, user association, access selection, routing, computation-aware resource management, and service provisioning [34,35,36,37]. These methods are valuable, but they are often evaluated with module-specific assumptions and metrics. As a result, it remains difficult to determine whether a gain in one function remains visible when the same scenario is passed through access, handover, routing, scheduling, and load-balancing modules. The platform-level gap addressed in this paper is therefore the lack of a traceable software-testbed workflow that treats algorithms as replaceable services consuming shared records and publishing comparable decision and metric outputs. Advanced machine learning (ML)-based intelligent control methods for STINs can also be integrated into such a workflow as plug-in engines [38]. In the present paper, however, the selected access, handover, routing, and scheduling methods are used as representative engines for validating the testbed interface rather than as definitive or state-of-the-art controllers. Table 1 summarizes the existing literature on simulation and validation approaches.

### 1.2. Contributions

To address the above gap, this study develops a modular software-based STIN testbed for platform design, algorithm integration, and performance emulation. The testbed is intended for controlled strategy evaluation rather than hardware-in-the-loop experimentation, field testing, or full packet-level NR-NTN protocol validation. Its design follows four principles: common scenario representation, explicit data contracts, replaceable functional engines, and traceable metric export. The main contributions are as follows.

- A modular STIN software-testbed architecture is developed to connect scenario generation, model-driven data processing, replaceable simulation engines, scheduler-based coordination, and Kafka-style message exchange.
- A unified data and message interface is defined through entity, link, traffic, decision, and metric records with scenario identifiers, timestamps, schema versions, and algorithm tags.
- A set of interoperable functional modules is specified for link-budget calculation, traffic generation, multi-attribute access, handover, scheduling, load balancing, adaptive routing, and multi-mode transmission.
- A lightweight performance-emulation model is implemented to evaluate access, handover, routing, and scheduling and to conduct parameter-sensitivity analyses in a 600-user reference STIN scenario.

The remainder of this paper is organized as follows. Section 2 presents the testbed architecture, data processing, and link-state modeling. Section 3 introduces the terrestrial and satellite functional modules. Section 4 presents the performance evaluation. Section 5 discusses the implications, limitations, and extension directions of the proposed testbed. Section 6 concludes the paper.

## 2. Testbed Architecture and Data Processing

**Figure 1:**
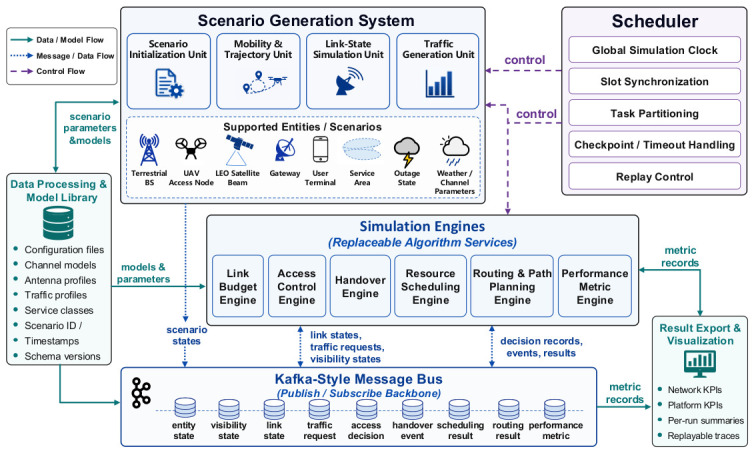
Block diagram of the proposed testbed architecture.

As shown in Figure 1, the proposed testbed consists of a scenario-generation system, simulation engines, a scheduler, and a Kafka-style publish/subscribe interface. The architecture represents terrestrial, aerial, and satellite entities in a common coordinate, time, and identifier space while separating scenario data from algorithm logic. It connects scenario construction, algorithm execution, data logging, and result visualization through explicit interfaces, thereby allowing an algorithm module to be replaced without regenerating the scenario state. The intended use cases include algorithm benchmarking, cross-domain resource orchestration, remote-sensor data delivery, emergency-communication planning, and software-level preparation for future hardware-in-the-loop evaluation.

### 2.1. Platform Workflow

The execution workflow is organized as a closed-loop simulation process, as illustrated in Figure 1. At the beginning of each run, the scenario generation system loads configuration files, entity parameters, mobility traces, traffic profiles, and channel settings. The scheduler initializes the global clock, partitions tasks by user group, region, beam, or domain, and advances a slot only after required engine outputs have been received or a configured timeout has been handled. Within each slot, the scenario generator publishes positions, visibility relationships, traffic requests, and environmental states, and the engines publish link quality, access, handover, scheduling, routing, and metric records. This phase separation prevents algorithms from reading partially updated states within the same slot. For long runs, checkpoints store the scenario identifier, slot index, random-seed state, engine-completion bitmap, and latest valid outputs from critical engines so that failed or delayed engines can be debugged or handled in degraded mode.

### 2.2. Implementation Principles

The platform is implemented around explicit data contracts rather than tightly coupled procedure calls. Each message contains a timestamp, a scenario identifier, entity identifiers, a payload type, payload data, and a schema version, thereby supporting slot-level synchronization and replay. Configuration files and model-library entries define reusable channel, antenna, mobility, satellite, traffic, and service-class parameters. Algorithm engines follow a common subscribe–process–publish interface. For example, a link-budget engine publishes received-power, SINR, delay, and feasible-link records, while an access engine consumes these records together with load-state and traffic-request records and then publishes serving-node decisions. This interface allows new algorithms to be evaluated without changing the scenario generator or global scheduler. The data contract used by the engines is summarized in Table 2. Here, the testbed denotes a software integration environment with explicit module interfaces rather than a physical experimental platform. The implementation follows the typed message interface, engine registration contract, and slot-level publish/subscribe procedure summarized in Table 2 and Table 3. The Kafka-style interface is represented by topic and partition metadata and replay offsets; the present study does not measure the throughput or fault tolerance of a deployed message broker. Table 4 summarizes the end-to-end emulation workflow.

### 2.3. Scenario Generation

The scenario generation system constructs and updates the network environment during each simulation run. The Scenario Initialization Unit loads terrestrial base stations (BSs), UAV-mounted access nodes, satellite-visibility or beam-center traces, gateway positions, user distributions, traffic profiles, and environment parameters. The Mobility and Trajectory Unit generates random-waypoint, vehicular, high-speed-rail, UAV, or satellite-visibility traces. The Link-State Simulation Unit updates large-scale path loss, shadowing, small-scale fading, atmospheric attenuation, rain attenuation, delay, and available bandwidth. The Traffic Generation Unit produces service requests with quality-of-service (QoS) attributes such as delay tolerance, packet size, reliability level, and service priority. These units provide the scenario initialization, mobility and trajectory, link-state simulation, and traffic-generation functions required by dynamic heterogeneous STIN emulation. The scenario generator supports both static and time-varying input sources. Static inputs describe base-station locations, satellite-visibility traces or beam centers, gateway positions, antenna parameters, transmit powers, and nominal bandwidth resources. Time-varying inputs describe user mobility, UAV trajectory, satellite visibility, weather-dependent attenuation, traffic arrivals, and temporary infrastructure outages. All scenario inputs are bound to a scenario identifier so that the same experiment can be regenerated or replayed later.

The model library provides reusable parameter sets for typical terrestrial, UAV, and satellite entity types. For a terrestrial node, the library stores carrier frequency, antenna height, transmit power, bandwidth, sector or beam identifier, and maximum access capacity. For a satellite beam, it stores altitude, the ground-projected beam center, antenna gain, propagation-delay baseline, feeder-link or gateway association, and beam-level capacity. For a service class, it stores packet-size distribution, traffic demand, priority, delay bound, and minimum rate requirement. These entries make the platform configurable without hard-coding algorithm assumptions in the simulation engines. The data-processing layer also maintains baseline adjacency matrices for terrestrial, satellite, gateway, and core-network domains. These matrices allow early reachability checks before higher-layer routing and scheduling algorithms are executed.

### 2.4. Simulation Engines

Simulation engines subscribe to scenario updates and execute independently or in parallel through common message schemas. The main engines and their interfaces are summarized in Table 5.

#### 2.4.1. Link-Budget Engine

The link-budget engine evaluates terrestrial cellular links, satellite links, UAV access links, and satellite–terrestrial feeder or service links under a unified interface. For each candidate link, the engine combines transmit power, antenna gain, carrier frequency, propagation distance, terrain class, fading state, atmospheric loss, rain attenuation, and interference margin to estimate received power, SINR, propagation delay, and feasible data rate. For a user *u* and candidate node *n*, the received power is represented in the dB domain as

(1) $$P_{u,n}^{r}=P_{n}^{t}+G_{n}^{t}+G_{u}^{r}-L_{u,n}^{\mathrm{path}}-L_{u,n}^{\mathrm{shadow}}-L_{u,n}^{\mathrm{atm}}-L_{u,n}^{\mathrm{rain}}.$$

Here, $P_{n}^{t}$ is transmit power, $G_{n}^{t}$ and $G_{u}^{r}$ are antenna gains, $L_{u,n}^{\mathrm{path}}$ is distance- and frequency-dependent path loss, and the remaining terms represent shadowing, atmospheric absorption, and rain attenuation. In the implemented emulation model, the basic free space path loss (FSPL) component is

(2) $$L_{u,n}^{\mathrm{FSPL}}=32.45+20\log_{10}\left(d_{u,n}^{\mathrm{km}}\right)+20\log_{10}\left(f_{n}^{\mathrm{MHz}}\right),$$

where $d_{u,n}^{\mathrm{km}}$ is the three-dimensional propagation distance and $f_{n}^{\mathrm{MHz}}$ is the carrier frequency. The path-loss term used by the lightweight emulator is $L_{u,n}^{\mathrm{path}}=L_{u,n}^{\mathrm{FSPL}}+L_{u,n}^{\mathrm{clutter}}$. The SINR abstraction is expressed in the dB domain as

(3) $$\gamma_{u,n}^{\mathrm{dB}}=P_{u,n}^{r}-N_{0}^{\mathrm{dBm}}-I_{u,n}^{\mathrm{dB}},$$

where $N_{0}^{\mathrm{dBm}}$ is the receiver noise floor and $I_{u,n}^{\mathrm{dB}}$ is the dB-equivalent interference margin. The noise floor is calculated as

(4) $$N_{0}^{\mathrm{dBm}}=-174+10\log_{10}\left(B_{\mathrm{Hz}}\right)+F_{N},$$

where $B_{\mathrm{Hz}}$ is the receiver bandwidth in hertz and $F_{N}$ is the receiver noise figure. The feasible rate is approximated as

(5) $$R_{u,n}=B\eta \left(\gamma_{u,n}^{\mathrm{dB}}\right),$$

where *B* is the available bandwidth and η(·) is the spectral-efficiency mapping used by the selected radio-access model. For the reported performance-emulation results, the spectral-efficiency mapping is

(6) $$\eta \left(\gamma \right)=\min\{6,\;0.78\log_{2}\left(1+10^{\gamma /10}\right)\},$$

which approximates adaptive modulation and coding without simulating packet-level PHY/MAC procedures. This comparative model does not emulate packet-level PHY/MAC behavior, Doppler compensation, or a 3GPP NR-NTN protocol stack. Its output is consumed by access, handover, routing, scheduling, and metric engines. The numerical parameters used in the performance evaluation are listed in Section 4.

#### 2.4.2. Access Control Engine

The access control engine performs candidate-node selection for terrestrial, satellite, and hybrid access. It supports received-signal-strength (RSS)-based selection, load-aware selection, and entropy-based multi-attribute selection. The common access interface exposes SINR, achievable rate, service priority, node load, idle resources, visibility duration, and backhaul availability. The evaluated access rules use explicit subsets of these fields as defined in Section 2.8; service priority is reserved for admission and scheduling rather than added as a candidate-invariant ranking term. For access control, the objective is to choose one serving node $a_{u}$ for each user while satisfying link-feasibility and node-capacity constraints. The access success rate is defined as

(7) $$\rho_{\mathrm{acc}}=\frac{\left|\{u:a_{u}\neq ⌀\}\right|}{\left|U\right|}.$$

The access engine can also record rejection causes such as insufficient received power, lack of satellite visibility, serving-node overload, and backhaul outage.

#### 2.4.3. Handover Engine

The handover engine monitors serving-link quality, neighboring-link quality, user mobility, satellite visibility, and expected dwell time. It supports intra-terrestrial handover, inter-satellite handover, and terrestrial–satellite handover. The triggering logic can combine event-level candidate changes, dwell-time estimation, and a lightweight swarm-guided candidate search to reduce ping-pong handovers. For a candidate target node, the handover engine evaluates candidate-node feasibility, expected remaining visibility, current-session delay budget, and target-node load before issuing a switching decision. The resulting handover record includes the source node, target node, trigger reason, interruption duration, candidate-switch acceptance flag, and ping-pong flag. The inputs to the swarm-guided mode are the candidate-link set, current serving node, previous serving node, last switching slot, node-load state, and visibility estimate. The outputs are a target-node decision and the associated handover-event record.

#### 2.4.4. Resource Scheduling Engine

The resource scheduling engine supports 4G/5G multi-user scheduling and satellite on-board resource scheduling. For terrestrial cells, it allocates time–frequency resources according to channel quality, instantaneous traffic demand, service priority, delay budget, and cell load. For satellite beams, it allocates time slots, frequency resources, and power resources according to beam load, the visible-user set, link-budget results, and gateway availability. The scheduling policy can be configured as equal allocation, proportional-fairness-inspired demand-normalized scheduling, utility-weighted scheduling, or a QoS-oriented heuristic scheduler for mission-oriented scenarios. In the utility-weighted policy used in the performance evaluation, the scheduling weight increases with service priority and decreases with traffic demand so that low-rate, delay-sensitive services are not starved by broadband flows. The QoS-oriented heuristic scheduler first admits users in descending order of a priority-aware utility-to-target-rate ratio and then distributes the remaining resources according to residual service utility. Its inputs are the served-user set, feasible rates, traffic demand, service priority, delay bound, and cell or beam budget. Its outputs are the per-user scheduled rate, QoS-satisfaction flag, utilization, throughput, and fairness records. For a cell or beam serving *M* users, sorting dominates the QoS-oriented scheduler with complexity O(MlogM), while the subsequent allocation scan is O(M). For a scheduling run, let $U^{\mathrm{served}}={⋃}_{n}U_{n}$ denote the users admitted by the fixed upstream association used in that run. The aggregate throughput is $\sum_{u\in U^{\mathrm{served}}}r_{u}$, where $r_{u}$ is the scheduled rate of user *u*. Fairness is measured by Jain’s fairness index:

(8) $$J=\frac{{\left(\sum_{u\in U^{\mathrm{served}}}r_{u}\right)}^{2}}{|U^{\mathrm{served}}|\sum_{u\in U^{\mathrm{served}}}r_{u}^{2}}.$$

QoS satisfaction is $\rho_{\mathrm{QoS}}=|\left\{u\in U^{\mathrm{served}}:r_{u}\geq \theta_{\mathrm{eval}}d_{u},\;D_{u}^{\mathrm{svc}}\leq \tau_{u}\right\}\left|/\right|U^{\mathrm{served}}|$, where $D_{u}^{\mathrm{svc}}$ is the instantaneous service-delay abstraction and $\tau_{u}$ is the service-delay bound. The denominator excludes users rejected before scheduling, so this metric is conditional on successful association and should not be compared directly with the access success rate. If $U^{\mathrm{served}}$ is empty, the metric is reported as not applicable rather than as zero. Each scheduling run builds the served-user set from access and link-state records, evaluates the selected policy, clips the scheduled rate to the feasible link capacity, and exports throughput, utilization, delay, fairness, and QoS-satisfaction records.

#### 2.4.5. Routing and Path Planning Engine

The routing engine constructs adjacency matrices for terrestrial access links, terrestrial backhaul links, gateway links, inter-satellite links, and satellite–terrestrial links. It determines feasible end-to-end paths in terrestrial-only, satellite-assisted, and adaptive cross-domain transmission modes. For a flow, blocking occurs when no feasible terrestrial, satellite-assisted, or hybrid path can satisfy reachability and service constraints. The path availability is $1-\rho_{\mathrm{blk}}$, where $\rho_{\mathrm{blk}}$ is the blocking rate. The path-cost interface can include propagation delay, queuing delay, congestion level, link reliability, switching penalty, gateway availability, and service priority. In the performance-emulation experiment reported in this study, the adaptive routing rule uses delay cost over feasible path candidates, while reliability is represented through feasibility and outage-state filtering. The remaining cost fields are retained as extensible interface entries for future routing algorithms.

#### 2.4.6. Performance Metric Engine

The performance metric engine aggregates algorithm outputs into network-level indicators, such as access success, candidate-switch acceptance, throughput, latency, blocking probability, QoS satisfaction, fairness, and transmission-mode utilization. It also reports platform-level indicators, such as message count, engine-completion status, and replay consistency. Each result record includes scenario identifiers, algorithm identifiers, random-seed tags, and timestamp ranges to prevent comparisons across inconsistent scenario states.

### 2.5. Scheduler and Kafka-Style Publish/Subscribe Interface

The scheduler maintains a slot table that records engine completion for each emulation time slot. An output is accepted only when its timestamp and scenario identifier match the active experiment. Delayed engines can be handled by timeout or by degraded execution using the last valid non-critical state. The scheduler also provides replay control, task partitioning, checkpoint management, and metric-aggregation triggers. Partition keys in message metadata allow downstream engines to merge partial results without losing scenario association. The scheduler coordination overhead comprises slot-completion checks, message validation, partition coordination, timeout handling, and checkpoint updates. For *E* registered engines, $M_{s}$ slot records, $P_{s}$ active partitions, and $T_{c}$ critical topics, its per-slot time complexity is $O(E+M_{s}+P_{s}+T_{c})$ and its checkpoint state is $O(E+P_{s})$, excluding engine-side computation. When the number of candidates per user and the number of topic types are bounded, $M_{s}$ grows linearly with the number of active users or flows; the coordination layer therefore scales linearly in the dominant record count.

The Kafka-style publish/subscribe interface uses topics, partition keys, offsets, consumer-group-style metadata, schema versions, and replay checkpoints. The present implementation evaluates this interface abstraction rather than benchmarking a deployed Kafka cluster [39]. Topics are organized by data semantics rather than by algorithm implementation. Typical topics include scenario state, entity position, visibility, link state, traffic request, access decision, handover event, scheduling result, routing result, and performance metric records. This topic design allows an engine to be replaced without changing the producers of its input data. For example, a new access algorithm can subscribe to the same link-state, load-state, and traffic-request topics as the baseline algorithm and publish decisions to the same access-decision topic with a different algorithm tag.

Partitioning is used to balance throughput and ordering requirements. Messages that belong to the same user, flow, beam, or region can be assigned to the same partition when strict local ordering is required. Messages that only contribute to aggregate metrics can be distributed across partitions to improve parallel processing. The scheduler controls global slot advancement, while offset metadata preserves local event order within each partition. Consumer-group-style metadata permits a future broker implementation to assign different partitions to multiple engine instances.

The interface includes metadata for replay, fault recovery, and external integration. Offsets identify restart positions, schema versions expose incompatible interface changes, and retention fields specify which records should remain available for offline comparison. These are software-interface provisions, not measurements of a deployed broker’s recovery or fault tolerance. The same record schemas can be consumed by visualization tools, external channel emulators, future hardware-in-the-loop interfaces, or offline analysis modules. Table 6 summarizes the Kafka-style publish/subscribe interface used by the software testbed.

### 2.6. Link-State Abstraction and Metric Definitions

The testbed uses a common link-state abstraction to represent terrestrial cellular, UAV, satellite service, satellite feeder, and inter-satellite links. This abstraction serves as a data contract for higher-layer algorithm comparison rather than implying that terrestrial, UAV, and satellite links share identical physical-layer behavior. Each link-state record describes the source entity, destination entity, domain tag, frequency band, bandwidth, propagation delay, received power, SINR, estimated rate, availability or reliability indicator, validity interval, optional beam identifier, remaining visibility duration, weather-loss terms, and optional Doppler-related state. The validity interval is important for satellite links because beam visibility and path delay change with satellite motion. The domain tag identifies whether the link belongs to terrestrial access, UAV access, satellite access, gateway, backhaul, or inter-satellite transmission. The actual link-state information, including received power, SINR, propagation delay, estimated rate, and reliability or availability, should first be computed from the link type, geometry, and propagation characteristics using the corresponding channel or link-budget model. For example, satellite–terrestrial and NR-NTN links can use channel-model assumptions such as those in 3GPP TR 38.811 [12], terrestrial cellular links can use channel models such as 3GPP TR 38.901 [40], and UAV or air-to-ground links can use appropriate aerial channel or link-budget models. The link-state abstraction is then formed from these computed results and packages them into a common record format for higher-layer decision engines. Doppler-related dynamics, beam variation, weather-dependent attenuation, and intermittent satellite visibility are exposed through optional fields and associated resource-state records. With these records, higher-layer engines can reject unavailable, unstable, or short-lived candidates without directly parsing orbital geometry, channel traces, or low-level radio states. More detailed terrestrial channel models, satellite-visibility traces, link-budget tools, or NR-NTN channel emulators can replace the link-state producer while preserving the same higher-layer interface. Packet-level PHY/MAC procedures and full NR-NTN protocol behavior remain outside the scope of the present abstraction.

The metric definitions are independent of the selected algorithm. For access, the platform reports success rate, average access delay, and load-balance index. For handover, it reports the candidate-switch acceptance rate, ping-pong ratio, and average interruption delay according to the selected handover model. For routing, it reports blocking rate, path availability, and average end-to-end delay. For scheduling, it reports aggregate throughput, QoS satisfaction, Jain’s fairness index, and bandwidth utilization. This common metric interface is necessary because a scheme that improves one dimension may degrade another.

### 2.7. Multi-Attribute Access Selection

The access engine includes an entropy-based multi-attribute decision-making method for heterogeneous candidate nodes. For candidate link *k* and attribute *f*, the raw attribute value $N_{k}^{f}$ is first transformed into a dimensionless benefit value ${\tilde{N}}_{k}^{f}$. For benefit attributes such as SINR and feasible rate, min–max normalization is applied so that larger values are preferred; for cost attributes such as load and delay, the normalized order is reversed. If all candidate values of an attribute are equal, its normalized values are set to one. When only one feasible candidate is available, the engine selects it directly and bypasses entropy weighting. For K≥2, where *K* is the number of feasible candidate links, the implementation forms

(9) $$q_{f}^{k}=\frac{\max({\tilde{N}}_{k}^{f},\delta_{q})}{\sum_{j=1}^{K}\max\left({\tilde{N}}_{j}^{f},\delta_{q}\right)},\;\delta_{q}=10^{-9}.$$

The entropy of the *f*-th attribute is

(10) $$\varphi_{f}=-\frac{1}{\ln K}\sum_{k=1}^{K}q_{f}^{k}\ln q_{f}^{k}.$$

The attribute weight is

(11) $$\varepsilon_{f}=\frac{\max(1-\varphi_{f},\delta_{\varepsilon })}{\sum_{g}\max\left(1-\varphi_{g},\delta_{\varepsilon }\right)},\;\delta_{\varepsilon }=10^{-6}.$$

The numerical floors prevent undefined logarithms and degenerate zero-weight denominators; an attribute with identical candidate values consequently receives only a floor-level weight. The entropy-weighted attribute score is $Q_{k}=\sum_{f}\varepsilon_{f}{\tilde{N}}_{k}^{f}$. The implemented serving-node score also includes the explicit load penalty defined in Section 2.8 to avoid repeatedly selecting overloaded nodes. Compared with fixed-weight selection, entropy weighting reduces manual parameter bias when candidate-specific link quality, delay, and load state vary across domains.

### 2.8. Algorithm Decision Rules Used in the Emulation

The replaceable algorithms evaluated in this study use the same candidate-link records and differ only in their decision rules. For a user *u*, let $C_{u}\left(t\right)$ denote the feasible serving-node set at slot *t*, and let $\ell_{n}\left(t\right)=N_{n}\left(t\right)/C_{n}$ denote the load ratio of node *n* with current assigned users $N_{n}\left(t\right)$ and abstract capacity $C_{n}$. The RSS-only baseline selects the node with the maximum received power.

(12) $$a_{u}^{\mathrm{RSS}}\left(t\right)=\arg\;\max_{n\in C_{u}\left(t\right)}P_{u,n}^{r}\left(t\right).$$

The load-aware access rule further incorporates the feasible rate and a capacity-normalized load penalty:

(13) $$a_{u}^{\mathrm{LA}}\left(t\right)=\arg\;\max_{n\in C_{u}\left(t\right)}\left[\gamma_{u,n}^{\mathrm{dB}}\left(t\right)+\alpha_{R}^{\mathrm{LA}}R_{u,n}\left(t\right)-\beta_{\ell }^{\mathrm{LA}}\ell_{n}\left(t\right)\right].$$

For entropy-based access, the decision score combines entropy-weighted normalized link attributes and an explicit load penalty:

(14) $$S_{u,n}^{\mathrm{EN}}\left(t\right)=\sum_{f}\varepsilon_{f}{\tilde{N}}_{u,n}^{f}\left(t\right)-\beta_{\ell }^{\mathrm{EN}}\ell_{n}\left(t\right).$$

The normalized attributes ${\tilde{N}}_{u,n}^{f}\left(t\right)$ characterize candidate-specific factors such as link quality, feasible rate, load state, and delay. The serving node is then selected as $a_{u}^{\mathrm{EN}}\left(t\right)=\arg\;\max_{n\in C_{u}\left(t\right)}S_{u,n}^{\mathrm{EN}}\left(t\right)$. $\alpha_{R}^{\mathrm{LA}}$, $\beta_{\ell }^{\mathrm{LA}}$, and $\beta_{\ell }^{\mathrm{EN}}$ are tunable weighting factors that control the relative importance of the corresponding decision attributes. User priority is retained in the traffic record for scheduling and admission policies but is not added as a candidate-invariant constant to this node-ranking score.

For handover decisions, the candidate target at slot *t* is derived from the same link-state and access-decision records. The threshold-only baseline immediately accepts a feasible serving-node change whenever the candidate target differs from the current serving node. By contrast, the dwell-time-aware rule accepts the candidate target only when the user has remained with its current serving node for at least a predefined dwell duration:

(15) $$h_{u}\left(t\right)=1\left\{a_{u}\left(t\right)\neq s_{u}\left(t\right),\;t-t_{u}^{\mathrm{last}}\geq T_{\mathrm{dwell}}\right\},$$

where $s_{u}\left(t\right)$ is the current serving node, $t_{u}^{\mathrm{last}}$ is the last switching slot, and $T_{\mathrm{dwell}}$ is a dwell-duration threshold. This threshold specifies the minimum number of slots that a user must remain with the current serving node before a new candidate handover can be accepted, thereby filtering short-lived candidate changes and reducing ping-pong handovers. A larger $T_{\mathrm{dwell}}$ improves handover stability but may delay or reject beneficial switches when the link condition changes quickly. The ping-pong metric uses an observation window that is independent of the dwell filter. Let $n_{u,k}$ denote user *u*’s serving node after its *k*-th accepted handover at slot $t_{u,k}$. The event indicator is

(16) $$z_{u,k}^{\mathrm{pp}}=1\left\{n_{u,k}=n_{u,k-2},\;0<t_{u,k}-t_{u,k-1}\leq T_{\mathrm{pp}}\right\}.$$

Thus, $T_{\mathrm{pp}}$ bounds the elapsed time from the preceding A→B switch to the return B→A switch. The reported ping-pong ratio is the number of such return events divided by the total number of accepted handovers.

The swarm-guided handover rule is implemented as a bounded discrete multi-particle fitness search over the same candidate set $C_{u}\left(t\right)$, as described in Algorithm 1. For candidate node *n*, the fitness score is

(17) $$\begin{array}{cc} F_{u,n}^{\mathrm{SW}}\left(t\right)= & w_{\gamma }^{\mathrm{SW}}{\bar{\gamma }}_{u,n}\left(t\right)+w_{R}^{\mathrm{SW}}{\bar{R}}_{u,n}\left(t\right)+w_{D}^{\mathrm{SW}}{\bar{D}}_{u,n}\left(t\right)\\ & +w_{\ell }^{\mathrm{SW}}\left(1-\ell_{n}\left(t\right)\right)+w_{V}^{\mathrm{SW}}V_{u,n}\left(t\right)-P_{u,n}^{\mathrm{sw}}\left(t\right)-P_{u,n}^{\mathrm{rev}}\left(t\right), \end{array}$$

where ${\bar{\gamma }}_{u,n}\left(t\right)$, ${\bar{R}}_{u,n}\left(t\right)$, and ${\bar{D}}_{u,n}\left(t\right)$ are normalized link-quality, rate, and delay-preference attributes, and $V_{u,n}\left(t\right)$ is the remaining-visibility score. The switching penalty is $P_{u,n}^{\mathrm{sw}}=0.10$ for a candidate different from the current node and zero otherwise; the immediate-reversal penalty is $P_{u,n}^{\mathrm{rev}}=0.25$ only when the candidate returns the user to its previous serving node within two slots. The vector $w^{\mathrm{SW}}=\left(w_{\gamma }^{\mathrm{SW}},w_{R}^{\mathrm{SW}},w_{D}^{\mathrm{SW}},w_{\ell }^{\mathrm{SW}},w_{V}^{\mathrm{SW}}\right)$ is a configurable handover-fitness profile. Because these attributes are normalized before scoring, the relative magnitudes of this vector express the intended decision priority in the lightweight emulator. The entries are scoring weights rather than probabilities and need not sum to one. All tested profiles use the same total attribute weight of 0.95 so that the scale of the attribute contribution remains comparable while the switching penalty, reversal penalty, and fitness-improvement threshold remain fixed. The swarm-guided search is not claimed as an optimized mobility-management protocol; it is included to validate that the testbed can host an iterative handover engine with explicit state, fitness, and stopping controls. For *U* users, at most *K* candidate nodes per user, *P* particles, and *I* iterations, normalization and candidate preparation require O(UK) work and particle updates require O(UPI) constant-time fitness evaluations. The resulting slot-level complexity is therefore $O\;\left(U(K+PI)\right)$.

For routing, each flow is evaluated over terrestrial-only, satellite-assisted, and hybrid path candidates. The terrestrial-only baseline accepts only paths whose terrestrial access and backhaul links remain feasible under the configured outage state. The satellite-assisted rule uses satellite service links only when the terrestrial path is unavailable. The adaptive cross-domain rule selects the feasible path with the lowest emulated end-to-end path delay after including terrestrial backhaul, satellite-hop, and cross-domain switching penalties.

(18) $$p_{f}^{★}=\arg\min_{p\in P_{f}^{\mathrm{feas}}}\left(D_{p}^{\mathrm{access}}+D_{p}^{\mathrm{backhaul}}+D_{p}^{\mathrm{switch}}\right).$$

This rule is intentionally simple because the purpose of the experiment is to verify cross-domain path substitution and metric export under common records rather than to optimize a full routing protocol. In the reported experiment, reliability enters routing through path feasibility and outage-state filtering, while load is indirectly reflected through the access and scheduling records that define available candidate paths.
**Algorithm 1** Swarm-Guided Handover Decision**Input:** Candidate links $C_{u}\left(t\right)$, serving node $s_{u}\left(t\right)$, previous node $s_{u}^{\mathrm{prev}}$, last switch slot $t_{u}^{\mathrm{last}}$, load ratios $\ell_{n}\left(t\right)$, fitness weights $w^{\mathrm{SW}}$, penalty parameters, $\Delta_{F}$, *P*, *I*, ω, $c_{1}$, and $c_{2}$**Output:** Target node $s_{u}^{★}\left(t\right)$ and handover flag $h_{u}\left(t\right)$  1:Normalize link-quality, feasible-rate, and delay-preference attributes over $C_{u}\left(t\right)$.  2:Initialize each particle position $x_{p}\in \left[0,K-1\right]$ and velocity $v_{p}$, where $K=|C_{u}\left(t\right)|$; initialize personal and global best states.  3:**for** i=1 to *I* **do**  4:      **for** p=1 to *P* **do**  5:            Draw $r_{1},r_{2}∼U\left[0,1\right]$ and set $v_{p}\leftarrow \omega v_{p}+c_{1}r_{1}\left(x_{p}^{\mathrm{best}}-x_{p}\right)+c_{2}r_{2}\left(x^{\mathrm{best}}-x_{p}\right)$.  6:            Set $x_{p}\leftarrow \mathrm{clip}\left(x_{p}+v_{p},0,K-1\right)$ and evaluate $F_{u,n}^{\mathrm{SW}}\left(t\right)$ at candidate index $\mathrm{round}(x_{p})$.  7:            Update the particle’s personal best position.  8:      **end for**  9:      Update the global best position.10:**end for**11:Set $s_{u}^{★}\left(t\right)$ to the global-best candidate; retain the current node if the candidate’s fitness improvement is below $\Delta_{F}=0.04$.12:**if** the selected node differs from $s_{u}\left(t\right)$ **then**13:      Set $h_{u}\left(t\right)\leftarrow 1$.14:**else**15:      Set $h_{u}\left(t\right)\leftarrow 0$.16:**end if**17:Publish the target node, trigger cause, interruption-delay estimate, and ping-pong flag.18:**return** $s_{u}^{★}\left(t\right)$ and $h_{u}\left(t\right)$.

For scheduling, users associated with the same serving node share an abstract cell or beam budget according to policy-dependent weights. Equal allocation uses $w_{u}=1$, the proportional-fairness-inspired demand-normalized baseline uses $w_{u}=1/\max\left(\delta_{d},d_{u}\right)$, and utility-weighted scheduling uses $w_{u}=p_{u}/\max\left(\delta_{d},d_{u}^{\rho_{U}}\right)$, where $d_{u}$ is the service demand, $\delta_{d}$ is a demand-floor parameter that prevents very small demands from producing excessively large scheduling weights, and $\rho_{U}$ controls demand compression in the utility baseline. The available slot budget is represented as $C_{n}^{\mathrm{slot}}=\min\left(\kappa_{C}\sum_{u\in U_{n}}R_{u,n},C_{\max}\right)$, where $\kappa_{C}$ and $C_{\max}$ are configurable resource-budget parameters. The provisional rate allocation is

(19) $$r_{u}=\min\left(d_{u},\;C_{n(u)}^{\mathrm{slot}}\frac{w_{u}}{\sum_{j\in U_{n(u)}}w_{j}}\right).$$

A served user is counted as QoS-satisfied when $r_{u}\geq \theta_{\mathrm{eval}}d_{u}$ and the emulated service delay is no greater than the service-delay bound. This definition makes unsatisfied cases in the stressed-load experiments demand-feasibility outcomes rather than calibrated service guarantees. A zero scheduled rate is an instantaneous unserved-demand indicator; it establishes neither permanent packet loss nor eventual service because queue carry-over, retransmission, and long-term delay accumulation are outside the lightweight model.

The QoS-oriented heuristic scheduler implements a lightweight priority-aware allocation rule for each serving node, as described in Algorithm 2. Let $U_{n}$ be the users served by node *n*, $C_{n}^{\mathrm{slot}}$ be the available abstract slot budget, $p_{u}$ be service priority, $d_{u}$ be demand, and $\tau_{u}$ be the delay bound. The rule admits target rates $r_{u}^{\mathrm{tar}}=\theta_{\mathrm{alloc}}d_{u}$ in descending order of the utility-to-target-rate ratio and then distributes the remaining capacity according to residual utility. The user utility used for target-rate admission is

(20) $$\eta_{u}=p_{u}\left(1+\frac{\lambda_{\tau }}{\max(\tau_{\min},\tau_{u})}\right).$$

Here, $\theta_{\mathrm{alloc}}$ is the scheduler’s admission target, whereas $\theta_{\mathrm{eval}}$ is the threshold used only by the QoS metric. The parameters $\lambda_{\tau }$, $\tau_{\min}$, and the residual-demand exponent $\rho_{\mathrm{res}}$ are specified in the performance-emulation setup. Users are considered in descending order of $\eta_{u}/r_{u}^{\mathrm{tar}}$. This rule approximates a mission-oriented scheduler that prioritizes delay-sensitive and high-priority flows when the offered load exceeds the available capacity. It is a heuristic emulation policy with explicit priority, target-rate, and capacity rules rather than a globally optimal full-packet scheduler. For a serving node with $M_{n}$ users, sorting dominates the scheduler and gives $O(M_{n}\log M_{n})$ complexity, so the total slot-level complexity is $O(\sum_{n}M_{n}\log M_{n})$ over all serving nodes.
**Algorithm 2** QoS-Oriented Heuristic Scheduler**Input:** Served-user set $U_{n}$, feasible rates $R_{u,n}$, demand $d_{u}$, priority $p_{u}$, delay bound $\tau_{u}$, resource budget $C_{n}^{\mathrm{slot}}$**Output:** Allocated rate $r_{u}$, QoS flag, utilization, throughput, and fairness records  1:Compute $r_{u}^{\mathrm{tar}}=\theta_{\mathrm{alloc}}d_{u}$ and utility $\eta_{u}=p_{u}\left(1+\lambda_{\tau }/\max\left(\tau_{\min},\tau_{u}\right)\right)$ for each $u\in U_{n}$.  2:Sort users in descending order of $\eta_{u}/r_{u}^{\mathrm{tar}}$.  3:Initialize $r_{u}=0$ and remaining budget $C_{\mathrm{rem}}=C_{n}^{\mathrm{slot}}$.  4:**for** each sorted user **do**  5:      **if** $C_{\mathrm{rem}}\geq r_{u}^{\mathrm{tar}}$ **then**  6:            Allocate $r_{u}=r_{u}^{\mathrm{tar}}$ and update $C_{\mathrm{rem}}$.  7:      **end if**  8:**end for**  9:Distribute residual budget over unsaturated users in proportion to $\eta_{u}/d_{u}^{\rho_{\mathrm{res}}}$.10:Clip $r_{u}$ by $d_{u}$ and the feasible link-rate bound $R_{u,n}$.11:Compute and publish throughput, QoS satisfaction, Jain’s fairness index, and utilization.

### 2.9. Data Processing and Model Management

Efficient data management is the basis of scalable STIN performance emulation. The testbed organizes data into a scenario-definition stage, a slot-update stage, and a result-export stage. The scenario-definition stage stores the baseline topology, user profiles, channel settings, gateway layout, and reusable model-library entries. The slot-update stage maintains the time-varying states required by the engines, including mobility, satellite-visibility or beam-center traces; link quality; traffic demand; and outage state. The result-export stage binds decisions and metrics to the scenario and algorithm tags so that later analysis can distinguish configuration changes from algorithm effects.

A Query ID association mechanism bridges logical and physical entities. Logical IDs identify network functions, users, services, or beams. Physical IDs identify platforms such as UAVs, satellites, gateway devices, or ground vehicles. The Query ID mechanism maps logical IDs to physical IDs so that an algorithm can retrieve position, trajectory, velocity, or beam state without changing its logical interface. This decoupling supports reassignment, such as migrating a base-station function from a ground node to a UAV or associating a logical beam with a different satellite during constellation motion.

Data consistency is maintained through schema validation, timestamp checking, scenario-ID checking, and unit normalization. The platform stores length, power, frequency, bandwidth, delay, and rate fields with explicit units so that terrestrial and satellite modules do not silently mix incompatible conventions. When a new algorithm is added, it only needs to satisfy the corresponding input and output schemas. This design reduces coupling between algorithm development and scenario construction.

## 3. Communication Simulation Functional Modules

This section maps the common engine interfaces to terrestrial, satellite, and cross-domain workflows.

### 3.1. Terrestrial Cellular Communication Modules

**Figure 2:**
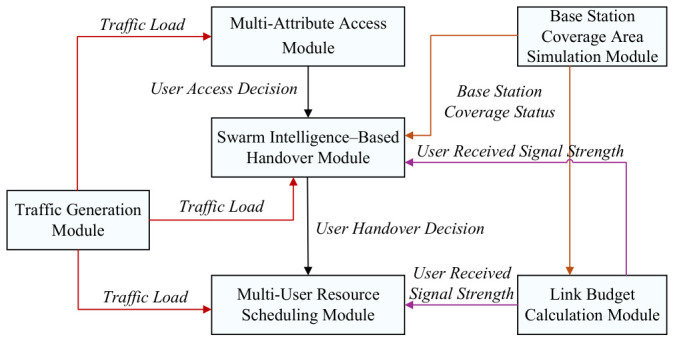
Flowchart of the terrestrial 4G/5G simulation algorithm modules.

The terrestrial workflow is organized in Figure 2 as a slot-synchronized loop linking traffic, link budget, access, handover, scheduling, and routing. Each module consumes the shared records defined in Section 2 and publishes tagged decisions or metrics.

#### 3.1.1. Coverage and Link Budget

The coverage module passes transmit power, antenna, position, frequency, environment, and trajectory parameters to the link-budget engine. In the reference configuration, a terminal is covered when RSSI >−110 dBm and SNR >−3 dB; these are emulation settings, not protocol constants. The resulting feasibility records constrain access, handover, and scheduling. The terrestrial fading and path-loss components are summarized in Table 7. The model library accommodates large-scale fading, shadow fading, atmospheric absorption, and small-scale fading effects that depend on the scenario and carrier frequency [23].

#### 3.1.2. Traffic and Scheduling

The traffic generator publishes service class, demand, arrival time, delay bound, and reliability fields using seed-controlled arrivals. Replaceable scheduling policies consume these requests and feasible-rate records, while the metric engine combines their outputs with the end-to-end path delays supplied by the routing engine.

#### 3.1.3. Terrestrial Handover and Routing

The handover policies operate on identical terrestrial, UAV, and satellite candidate records; the swarm rule adds a fitness search over link quality, load, visibility, and reversal penalties. The routing engine builds access and backhaul adjacency from reachability and gateway states and requests satellite-assisted candidates when terrestrial backhaul fails.

### 3.2. Satellite Communication Modules

**Figure 3:**
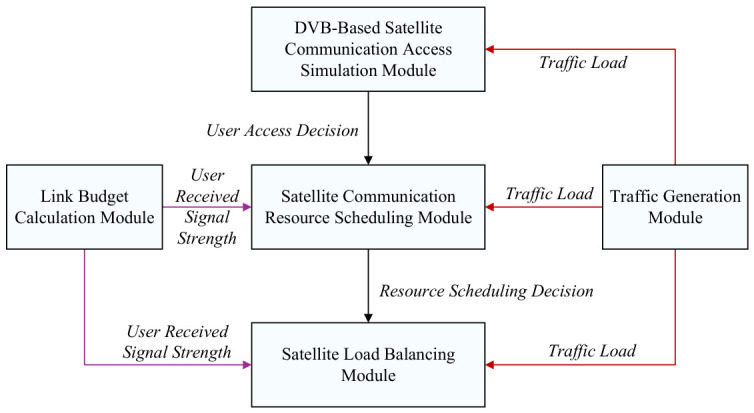
Flowchart of the satellite communication simulation algorithm modules.

Figure 3 links traffic generation, beam access, link quality, scheduling, and load balancing. The satellite access interface exposes SINR, feasible rate, visibility, available resources, and service priority, after which the scheduler allocates the abstract beam budget.

#### 3.2.1. Satellite Access and Resource Scheduling

The Digital Video Broadcasting (DVB)-inspired abstraction represents satellite framing and resource allocation, not an NR-NTN protocol stack [41]. In the evaluated entropy-based rule, visibility and backhaul state first determine the feasible beam set, after which the visible beams are ranked using candidate-specific link quality, feasible rate, load, and delay attributes [36]. Service priority remains available to the admission and scheduling engines but is not added as a candidate-invariant ranking term. The access engine then publishes the selected beam, access delay, or rejection cause. The beam scheduler applies the same replaceable allocation policies as the terrestrial workflow and exports rates, delay, utilization, and continuity metrics [16,35].

#### 3.2.2. Satellite Load Balancing and Link Budget

**Figure 4:**
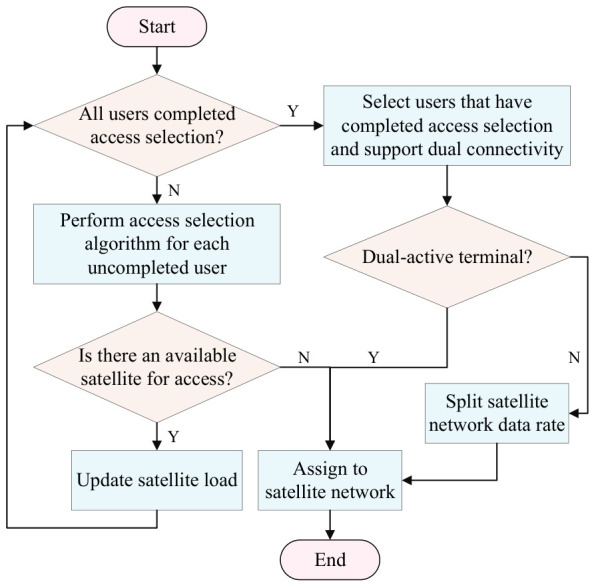
Flowchart of the satellite load-balancing procedure.

The load-balancing interface can reassign service flows among visible beams according to link quality and load. Figure 4 details the satellite load-balancing procedure.

The satellite–terrestrial link-budget engine evaluates satellite-to-terminal, terminal-to-satellite, satellite-to-gateway, and inter-satellite links. It accounts for free-space loss, atmospheric absorption, scintillation, rain attenuation, cloud attenuation, antenna losses, and fading effects [42,43]. The satellite traffic-generation interface can represent periodic, random, or burst-oriented service requests for sensor telemetry, broadband access, emergency communication, and delay-sensitive services [44]. The reported experiments in Section 4, however, use seed-controlled demand samples without time-correlated burst arrivals or inter-slot queue evolution. The multi-mode transmission module supports terrestrial-only transmission, satellite-only transmission, satellite-assisted fallback, and adaptive cross-domain routing. Mode selection is driven by the same link-state and traffic-demand records used by the access and scheduling engines, which keeps the satellite and terrestrial domains consistent during evaluation.

### 3.3. Cross-Domain Coupling Among Modules

Record-level coupling distinguishes the testbed from independent module runs: access determines active links, scheduling changes load, and routing consumes access and backhaul states. The scheduler preserves temporal order, while the cross-domain engine maps link records to a common path graph and returns path delay, availability, and load indicators. Consequently, each policy can be assessed by both its local decision and its end-to-end effect.

## 4. Performance Evaluation

### 4.1. Evaluation Setup

The evaluation objective is to verify whether the proposed software testbed supports integrated STIN modeling, replaceable algorithm execution, multi-mode emulation, and repeatable assessment. Table 8 lists the parameters used in the performance evaluation. The carrier frequency, altitude, link-budget, and NTN abstraction choices are based on standards-informed assumptions and values reported for NR-NTN studies, LEO link-budget analysis, and scenario-consistent channel modeling [12,13,14,23,42,43]. The traffic profiles are lightweight service-demand abstractions used to emulate mixed sensing, remote-IoT, broadband, and emergency traffic rather than packet-level application traces [44]. In the reference service mix, mMTC users represent periodic or event-triggered remote sensors with low rates and relaxed delay constraints; URLLC users represent alarm or emergency monitoring traffic with tighter latency requirements; and eMBB users represent high-volume sensing-data uploads or broadband backhaul traffic. The evaluation tests common-scenario execution, multi-dimensional metric export, and consistent numerical interpretation under fixed emulation settings. Unless otherwise stated, the plotted results report the mean value over seven deterministic replicas derived from the base seed, and the shaded regions or error bars denote one sample standard deviation.

The values in Table 8 are emulation parameters chosen to preserve the relative behavior of terrestrial, UAV, and satellite links rather than calibrated measurements of a specific deployed network. The LEO beam nodes in this table are ground-projected beam-level abstractions used for coverage, link-budget, and load evaluation. They should not be read as the number of physical satellites in an orbital constellation. The reference topology is designed as a controlled stress scenario. In this setting, five macro BSs, two UAV nodes, and four ground-projected LEO beam nodes jointly serve the 100km×100km area, providing terrestrial, aerial, and satellite access options while ensuring that fallback and beam-level load constraints are exercised. This compact topology is representative only of a sparse cross-domain stress case, not of a particular operator deployment. The 20 GHz satellite carrier represents a Ka-band downlink service link. The 0.2–2.8 dB random rain term represents light-to-moderate excess loss rather than a calibrated regional rain-rate model; the separate sensitivity sweep extends the range to 5–12 dB to test stronger attenuation, but does not replace an ITU-R or site-specific propagation calculation. The local sensitivity settings are specified explicitly for reproducibility. The load-aware penalties are 45, 60, 75, 85, 95, 110, and 125, and the entropy-load coefficients are 0.10, 0.20, 0.30, 0.35, 0.40, 0.50, and 0.60. The link-quality-emphasis profiles $\left(w_{\gamma },w_{R},w_{D},w_{\ell },w_{V}\right)$ are Q3=(0.38,0.30,0.10,0.10,0.07), Q2=(0.34,0.28,0.12,0.13,0.08), and Q1=(0.31,0.25,0.15,0.15,0.09). The reference profile is Ref.=(0.28,0.23,0.17,0.17,0.10). The stability-emphasis profiles are S1=(0.25,0.21,0.17,0.18,0.14) and S2=(0.22,0.18,0.18,0.19,0.18). The final profile is S3=(0.20,0.16,0.19,0.20,0.20). Each profile has a total attribute weight of 0.95. The dwell sweep uses one through seven slots, and the QoS target factors are 0.65, 0.75, 0.80, 0.85, 0.90, 0.95, and 1.00. The rain-loss ranges are R0=0.0–0.5, R1=0.0–1.0, R2=0.2–2.8, R3=1.0–4.0, R4=2.0–6.0, R5=3.0–8.0, and R6=5.0–12.0 dB. The evaluation scenario uses a two-dimensional service area, simplified LEO visibility, stochastic user locations, service-dependent traffic demand, dB-domain link-budget approximation, and deterministic terrestrial backhaul outage regions. The base random seed and its deterministic replicas reuse the same user set, candidate-link set, traffic profile, mobility trace, and backhaul-outage state across compared algorithms. The access, handover, routing, and scheduling sweeps vary user density, mobility scale, outage intensity, and demand multiplier, respectively. The results should therefore be interpreted as controlled performance emulation for workflow consistency, algorithm replaceability, and metric reporting under remote-sensor, remote-IoT, and emergency-service assumptions.

### 4.2. Evaluated Algorithms and Metrics

The evaluated algorithms are representative plug-in engines used to validate the software testbed rather than definitive or globally optimal policies. They are included to verify that heterogeneous control engines can consume shared scenario records, interact with the same execution workflow, and publish comparable metrics with consistent scenario and algorithm tags. Advanced ML-based algorithms can also be registered as algorithm modules and invoked by the platform scheduler or orchestration layer through the same interface. However, the design, training, and implementation of ML-based policies are beyond the scope of this paper, which focuses on the testbed architecture and its ability to support replaceable strategy evaluation. The access evaluation compares RSS-only, load-aware, and entropy-based access. RSS-only selects the node with the strongest received signal among feasible candidates. Load-aware access introduces a load penalty so that capacity-limited nodes are not repeatedly selected. Entropy-based access jointly evaluates candidate-specific link quality, feasible rate, delay, and load state through the multi-attribute scoring mechanism described in Section 2.7. The reported metrics are access success rate, average access delay, and load-balance index.

The handover evaluation compares threshold-only, dwell-time-aware, and swarm-guided event filtering. Threshold-only handover accepts a feasible serving-node change whenever the candidate target differs from the current serving node. Dwell-time-aware handover adds a minimum residence-time requirement to avoid unstable switching. Swarm-guided handover applies Algorithm 1 to the same candidate records and evaluates target-link quality, load, visibility, and reversal penalty through a discrete swarm search. The reported metrics are candidate-switch acceptance rate, ping-pong ratio, and average handover delay.

The routing evaluation compares terrestrial-only, satellite-assisted, and adaptive cross-domain routing. Terrestrial-only routing uses only terrestrial access and backhaul paths. Satellite-assisted routing uses satellite resources when the terrestrial path is unavailable. Adaptive cross-domain routing evaluates terrestrial, satellite, and mixed paths by minimizing an emulated path-delay cost over feasible candidates. The reported metrics are blocking rate, path availability, and average end-to-end delay.

The scheduling evaluation compares equal allocation, proportional-fairness-inspired demand-normalized scheduling, utility-weighted scheduling, and QoS-oriented heuristic scheduling. All scheduling policies consume the same served-user set and feasible-link records within a replica; QoS satisfaction is therefore conditional on this fixed upstream association. Equal allocation provides a simple capacity-sharing baseline. The proportional-fairness-inspired baseline uses demand-normalized weights as a lightweight fairness-oriented rule rather than a full historical-throughput proportional-fairness scheduler. Utility-weighted scheduling favors high-priority or lower-demand flows that are more likely to meet their QoS targets under heterogeneous traffic demands. QoS-oriented heuristic scheduling applies Algorithm 2 to admit mission-oriented target rates before distributing residual capacity. The reported metrics are throughput, QoS satisfaction, Jain’s fairness index, and bandwidth utilization. The concrete decision rules used in these comparisons are defined in Section 2.8.

### 4.3. Access and Handover Results

**Figure 5:**
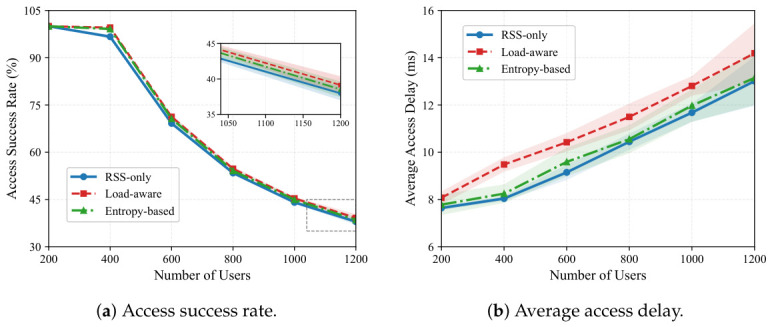
Access-control performance under increasing user density.

Figure 5 shows how access policies respond to growing user density. At 600 users, RSS-only, load-aware, and entropy-based access serve 69.17%±0.54%, 71.24%±0.64%, and 70.64%±1.01% of users, respectively. Load-aware access gives the highest admission rate because its explicit congestion penalty most strongly avoids saturated nodes; entropy-based access instead balances congestion with rate and delay, yielding lower delay than load-aware access (9.59±0.61 versus 10.42±0.39 ms) at a small admission cost.

**Figure 6:**
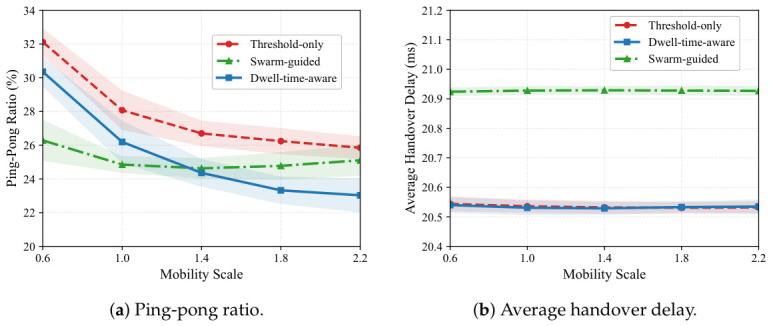
Handover performance under increasing mobility scale.

Figure 6 compares all policies on identical movement and visibility records using the common window $T_{\mathrm{pp}}=10$ slots. At the reference mobility point, threshold-only, dwell-time-aware, and swarm-guided handover yield ping-pong ratios of 28.07%±1.17%, 26.19%±1.27%, and 24.85%±0.49%, with mean delays of 20.54±0.02, 20.53±0.03, and 20.93±0.02 ms, respectively. The dwell filter accepts only 38.82%±0.32% of candidate switches because changes within three slots are rejected; its modest stability gain therefore comes at a substantial responsiveness cost. The swarm rule further suppresses reversals through joint load, visibility, and switching penalties, at the expense of a small emulated search delay.

### 4.4. Routing and Scheduling Results

**Figure 7:**
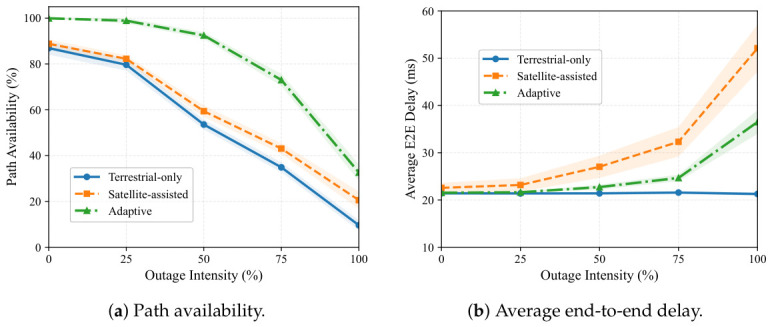
Cross-domain routing performance under increasing terrestrial backhaul outage intensity.

As shown in Figure 7, at a terrestrial backhaul-outage intensity of 50%, terrestrial-only, satellite-assisted, and adaptive routing provide 53.57%±2.16%, 59.40%±2.51%, and 92.47%±1.45% path availability, respectively. Satellite fallback improves reachability but raises delay to 27.04±2.37 ms because of the longer satellite path; adaptive routing limits this cost to 22.74±0.52 ms by selecting satellite or mixed paths only when they provide a feasible, lower-cost alternative.

**Figure 8:**
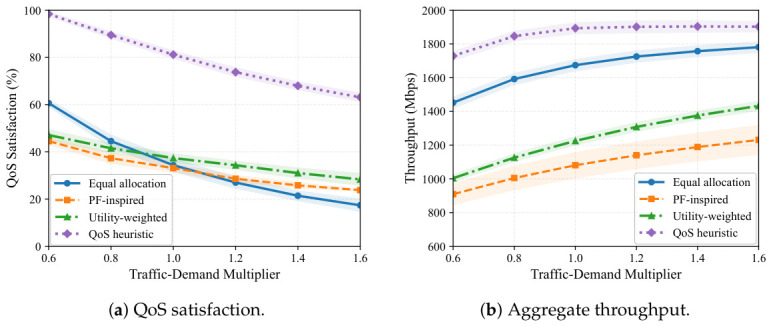
Scheduling performance under increasing traffic-demand multiplier.

As shown in Figure 8, at the reference demand multiplier of 1.0, equal, PF-inspired, utility-weighted, and QoS-oriented scheduling attain 34.37%±2.91%, 33.13%±1.94%, 37.38%±2.04%, and 81.19%±1.78% instantaneous QoS satisfaction, respectively. The QoS-oriented rule also delivers 1893.32±35.63 Mbps, but Jain’s fairness index falls to 0.6365±0.0157 because target-rate admission concentrates resources on high-utility flows. The remaining 18.81% of served users fail the instantaneous QoS criterion under the stressed mixed-service load, predominantly because the scheduled rate is below target. For the QoS-oriented policy, only four of the 2413 rate-feasible user instances across the seven replicas fail the delay bound. In practice, the shortfall would require admission control, traffic shaping, service slicing, or additional cell/beam capacity. Thus, the gain is a QoS–fairness tradeoff under controlled instantaneous demand, not a deployment-level service guarantee.

### 4.5. Sensitivity Analysis

The sweep experiments in Figure 5, Figure 6, Figure 7 and Figure 8 provide a compact sensitivity analysis under workload, mobility, outage, and demand variations. In the workload dimension, the access-density results in Figure 5 show full access for all policies at 200 users, followed by declining success rates once the offered user population exceeds the abstract node capacities. At 1200 users, the access success rates of RSS-only, load-aware, and entropy-based access decrease to 37.98%±0.99%, 39.14%±1.25%, and 38.54%±1.11%, respectively. This trend shows that the access module exposes capacity saturation rather than masking overload by always attaching users to the strongest link. Load awareness gives the largest success-rate benefit under heavy congestion, whereas entropy-based access maintains a lower delay than load-aware access across the density sweep.

For mobility sensitivity, Figure 6 isolates the effect of dwell-time filtering on handover stability. As the mobility scale increases from 0.6 to 2.2, the threshold-only ping-pong ratio decreases from 32.12%±0.81% to 25.85%±0.68%, and the dwell-time-aware ratio decreases from 30.36%±0.86% to 23.03%±1.02%. The swarm-guided ratio changes from 26.29%±1.21% to 25.11%±0.92% over the same sweep and is lowest at the reference mobility point. Its delay curve remains close to 20.9 ms because the emulator adds a fixed control-search cost to the selected handover events. These results show that the relative ordering is mobility-dependent and that none of the policies eliminates return events under the independent 10-slot observation window.

Under terrestrial backhaul disruption, the outage sweep in Figure 7 evaluates the benefit of satellite assistance as terrestrial reachability degrades. At zero outage intensity, the adaptive rule records 99.95%±0.15% path availability because it can use all feasible terrestrial, satellite, and mixed candidates. When outage intensity reaches 100%, the path availabilities of terrestrial-only, satellite-assisted, and adaptive routing are 9.62%±2.24%, 20.55%±3.77%, and 32.64%±3.67%, respectively. The corresponding adaptive delay increases from 21.54±0.12 ms to 36.51±2.52 ms, reflecting the expected cost of using longer cross-domain paths under severe terrestrial disruption.

The demand-side sensitivity in Figure 8 further clarifies the scheduling tradeoff for heterogeneous sensor and broadband traffic. When the demand multiplier rises from 0.6 to 1.6, QoS satisfaction under QoS-oriented heuristic scheduling decreases from 98.38%±1.12% to 63.13%±1.99%, while that under utility-weighted scheduling decreases from 47.12%±2.52% to 28.33%±2.76%. The QoS-oriented heuristic policy therefore gives the highest QoS-satisfaction rate throughout the demand sweep, although its admission-oriented allocation produces a lower value of Jain’s fairness index than the simpler sharing policies. Together, these sweeps support controlled strategy comparison under remote-sensor, remote-IoT, broadband, and emergency-service assumptions.

**Figure 9:**
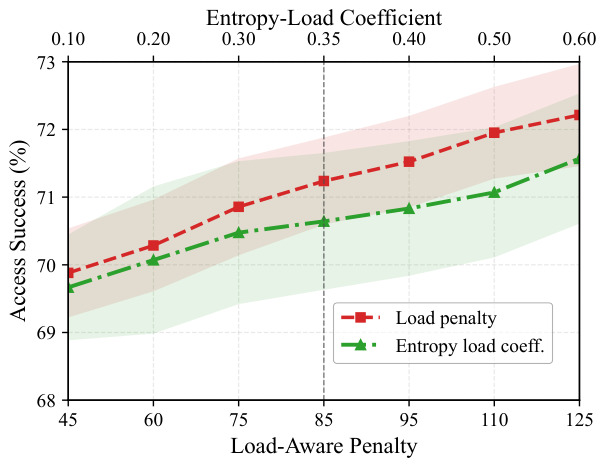
Access-coefficient sensitivity. The bottom axis varies the access load penalty; the top axis varies the entropy-load coefficient.

**Figure 10:**
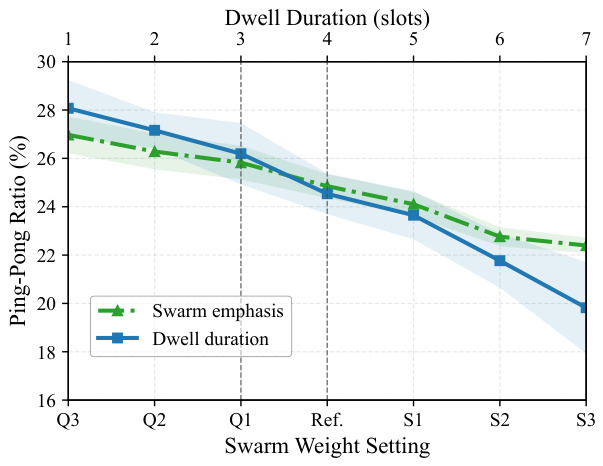
Handover sensitivity. The bottom axis shows the swarm weight profiles; the top axis shows the dwell duration.

Local robustness is examined by perturbing the reference parameters under controlled scenario settings. Except for the coupled QoS-target experiment discussed below, each sweep varies one parameter family while holding the remaining parameters fixed. The same seven seeds are reused at every point, and the shaded bands denote one sample standard deviation. For access control, Figure 9 indicates that increasing either the load-aware penalty or the entropy-load coefficient yields a modest improvement in admission over the evaluated range. Read together with the delay results in Figure 5, this trend illustrates the underlying tradeoff: stronger load suppression avoids saturated nodes, but some users are consequently redirected to links with longer delays. Two complementary stability mechanisms are compared in Figure 10. Moving the swarm profile from link-quality emphasis (Q3, Q2, Q1) through the reference profile toward stability emphasis (S1, S2, S3) lowers the ping-pong ratio from 26.97% to 22.40%, with little change in mean handover delay. A longer dwell requirement has an even stronger filtering effect: increasing the duration from one to seven slots reduces the ratio from 28.07% to 19.82%. This improvement is not cost-free, however, because the corresponding run records show a decrease in the mean number of accepted handovers from 6493.1 to 1129.7. Thus, greater stability is achieved by sacrificing responsiveness to candidate-link changes.

**Figure 11:**
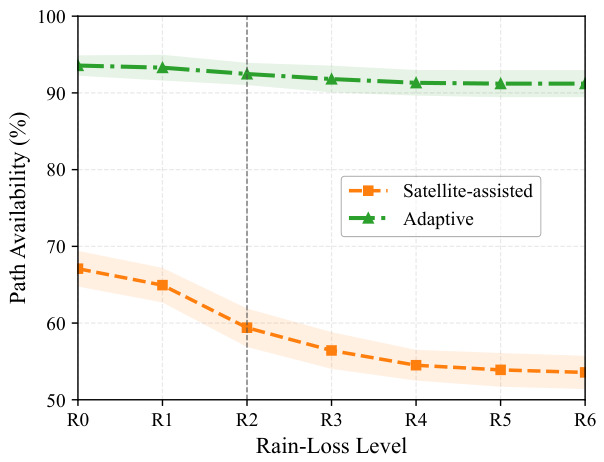
Effect of rain loss on routing performance.

**Figure 12:**
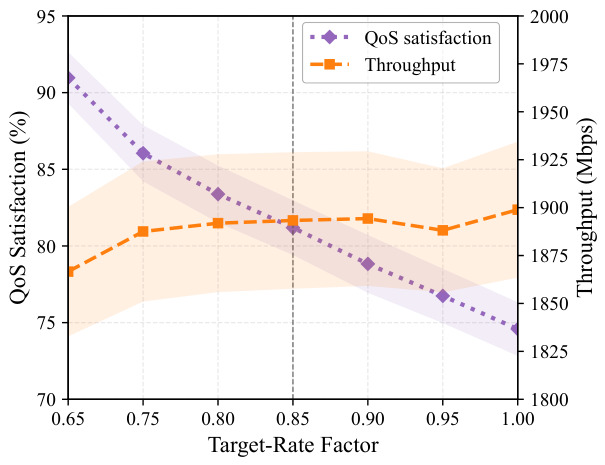
Joint sensitivity to the QoS target, with $\theta_{\mathrm{alloc}}=\theta_{\mathrm{eval}}$ at each sweep point.

Rain attenuation produces a different pattern in Figure 11. Satellite-assisted path availability declines as the excess loss increases, whereas adaptive routing remains above 91% throughout the sweep. Its weaker sensitivity follows from the availability of terrestrial and mixed alternatives when satellite links deteriorate. The scheduling experiment in Figure 12 requires a more careful interpretation. As $\theta_{\mathrm{alloc}}$ and $\theta_{\mathrm{eval}}$ increase jointly, QoS satisfaction declines, yet aggregate throughput changes only slightly under the fixed capacity budget. Because both the admission target and the evaluation threshold are tightened simultaneously, the resulting curve measures sensitivity to a stricter end-to-end service target; it should not be interpreted as an isolated robustness test of either scheduler parameter.

## 5. Discussion

The principal value of the proposed testbed is the ability to compare heterogeneous control strategies using common scenario states, data contracts, and metric definitions. Within this workflow, the experiments expose consistent tradeoffs between admission and delay, stability and responsiveness, reachability and propagation cost, and QoS satisfaction and fairness. The contribution therefore concerns traceable integration and comparison rather than the optimality of the evaluated heuristics.

The results remain bounded by a software-only, link-level emulator. Although the abstraction retains domain-dependent coverage, delay, visibility, capacity, demand, and outage effects, it omits packet-level PHY/MAC procedures, detailed interference and retransmissions, protocol timing, measured orbital and beam dynamics, and time-correlated channels. The compact topology and generated inputs are not deployment-calibrated, while the 30-slot handover traces restrict conclusions about long-term mobility. Moreover, the uncertainty bands represent only the stochastic terms included in the model, not model uncertainty or correlated traffic. Without inter-slot queues, a zero scheduled rate denotes instantaneous unserved demand rather than permanent packet loss. The reported metrics should therefore be read as comparative indicators, not deployment predictions.

A separate limitation concerns implementation. The Kafka-style publish/subscribe component defines message and replay interfaces but is not a deployed distributed broker; accordingly, the analytical complexity results do not establish throughput, fault recovery, hardware runtime, or wall-clock scalability. Larger networks will require validation with candidate pruning, partitioning, and parallel engine instances.

Future validation should progress from improving model fidelity to implementing the complete system. Generated inputs should first be replaced with calibrated deployment data and measured traces, followed by the integration of packet queues, retransmissions, and protocol evolution. The final stage should evaluate distributed-broker performance, joint cross-module policies, ML-based engines, and hardware-in-the-loop interfaces under the same data contracts.

## 6. Conclusions

This study presented a software-based modular testbed design and performance-emulation framework for satellite–terrestrial integrated networks. The platform integrates scenario generation, model-driven data processing, replaceable simulation engines, scheduler-based process control, and a Kafka-style publish/subscribe interface into one experimental workflow. The terrestrial and satellite modules support link-budget calculation, access control, mobility-aware handover, traffic generation, resource scheduling, satellite load balancing, adaptive path planning, and multi-mode transmission emulation. A lightweight model with deterministic replicas was used to evaluate access, handover, routing, and scheduling strategies and their parameter sensitivity. The results expose tradeoffs among access success rate, handover stability, path availability, QoS satisfaction, throughput, and fairness under controlled assumptions. These capabilities are relevant to sensing-oriented and remote-IoT STIN services because periodic monitoring, event-triggered alarms, telemetry collection, and high-volume sensing-data upload require coordinated evaluation of coverage extension, service continuity, routing resilience, and heterogeneous resource allocation. Future work will focus on packet-level protocol integration, ML-based policy integration, and hardware-in-the-loop performance evaluation.

## Figures and Tables

**Table 1:** Comparison of representative simulation and validation approaches.

Approach	Main Strength	STIN Integration	Replaceable Algorithms	Traceable Performance Metrics
ns-3/OPNET-style network simulation [26,27]	Packet/protocol modeling and mature network abstractions	Cross-domain STIN workflows usually require additional extensions	Possible but often scenario-specific	Metric export depends on custom instrumentation
Satellite-network simulators [28,29]	Constellation visibility and satellite link modeling	Strong satellite-domain support; terrestrial coupling often needs extension	Usually focused on selected functions	Depends on simulator implementation
Digital-twin-oriented STIN studies [30,31]	Virtual modeling and closed-loop optimization concepts	Strong potential when calibrated models and interfaces are available	Depends on twin-interface implementation	Often application- or scenario-dependent
Technology-specific satellite/IoT testbeds [33]	Implementation-oriented design and validation	Strong for the selected backhaul technology and deployment	Usually tied to the implemented protocol and hardware	Physical or protocol-specific measurements
Algorithm-specific studies [16,35,36,37]	Focused optimization of access or scheduling	Usually abstract or single-module evaluation	Usually not packaged as a shared testbed interface	Usually reports algorithm metrics only
Proposed software testbed	Closed-loop STIN platform with data contracts and scheduler	Terrestrial, satellite, and hybrid modes share scenario and metric spaces	Access, handover, scheduling, routing, and load balancing are replaceable	Network and platform metrics are exported with scenario and algorithm tags

**Table 2:** Core data objects exchanged by platform modules.

Data Object	Mandatory Fields	Main Producer	Main Consumer
Entity state	Scenario ID, timestamp, logical ID, physical ID, position, velocity, entity type	Scenario generator and mobility model	Link budget, visibility, access, and handover engines
Link state	Scenario ID, timestamp, user ID, node ID, distance, received power, SINR, rate, delay	Link-budget engine	Access, handover, routing, scheduling, and metric engines
Traffic request	Scenario ID, timestamp, source, destination, service class, demand, delay bound, priority	Traffic generator	Access, routing, scheduling, and QoS metric engines
Decision record	Scenario ID, timestamp, algorithm ID, selected node or path, status, failure reason	Access, handover, routing, scheduling, and load-balancing engines	Scheduler, metric engine, replay module, and visualization module
Metric record	Scenario ID, timestamp, metric name, aggregation scope, value, unit, algorithm tag	Performance metric engine	Result exporter, visualization service, and offline analysis tools

**Table 3:** Representative interfaces and schemas used by the software testbed.

Interface	Example Interface or Topic	Required Fields	Function in the Workflow
Engine registration	engine.register	Engine ID, consumed topics, produced topics, schema version, timeout policy	Allows the scheduler to discover replaceable engines and verify slot completion
Scenario publication	scenario.entity_state	Scenario ID, slot index, entity ID, entity type, position, velocity, timestamp, unit tags	Provides synchronized geometry and mobility states to link-budget and access engines
Link-state schema	link.state	Scenario ID, slot index, user ID, node ID, domain tag, distance, received power, SINR, rate, delay, valid flag	Exposes a common terrestrial, UAV, and satellite candidate-link record
Decision schema	decision.access	Scenario ID, slot index, algorithm ID, user ID, selected node, status, failure reason, seed tag	Separates algorithm-specific decisions from shared scenario and link-state generation
Metric query	metric.query	Scenario ID, algorithm ID, metric name, time range, aggregation scope, seed tag	Retrieves comparable network and workflow indicators for analysis and plotting

**Table 4:** Representative end-to-end emulation workflow.

Stage	Consumed Records	Engine Action	Published Output
Scenario setup	Configuration, seed, topology, service profile	Generate users, nodes, beam centers, mobility, traffic, and outage state	Entity state, traffic request, visibility state, outage state
Link-state update	Entity state, channel model, antenna profile, weather state	Compute received power, SINR, feasible rate, delay, and link validity	Link-state and feasible-link records
Algorithm execution	Link state, traffic request, node load, topology state	Run tagged access, handover, routing, and scheduling policies	Decision records, path records, handover events, scheduled rates
Slot coordination	Engine outputs, timestamps, partition keys, offsets	Check slot consistency, update checkpoints, and trigger aggregation	Engine-completion bitmap and replay offsets
Metric export	Decision records, link states, traffic records	Aggregate metrics with scenario, seed, and algorithm tags	Per-run summaries, curve points, and replayable metric records

**Table 5:** Input and output interfaces of main simulation engines.

Engine	Main Inputs	Main Outputs	Time Scale
Link budget	Entity states, channel parameters, antenna parameters, weather state, interference margin	Received power, SINR, feasible rate, propagation delay, feasible-link flag	Slot-level
Access control	Candidate links, node load, service priority, backhaul status, capacity constraints	Serving node, access status, access delay, failure reason	Slot- or event-level
Handover	Serving link, neighboring links, velocity, visibility duration, dwell timer	Handover trigger, target node, handover delay, ping-pong flag	Event-level
Scheduling	Access results, instantaneous traffic requests, rate estimates, QoS requirements, resource budgets	Allocated bandwidth, scheduled rate, emulated service delay, utilization, fairness	Slot-level
Routing and path planning	Domain topology, link reliability, load state, gateway state, traffic demand	Terrestrial, satellite-assisted, or hybrid path, blocking status, end-to-end delay	Flow-level
Metric aggregation	Algorithm decisions, link states, traffic records, scheduler traces	Network metrics, platform metrics, per-algorithm summaries, replay records	Slot- and-run level

**Table 6:** Kafka-style publish/subscribe interface design.

Design Element	Platform Role	Typical Configuration	Benefit
Topic taxonomy	Separates scenario state, link state, decisions, and metrics	Domain-specific topics with schema-version headers	Enables independent algorithm replacement
Partition key	Controls local ordering and parallelism	User ID, flow ID, beam ID, region ID, or domain ID	Balances slot consistency and scalability
Consumer group	Describes assignment to multiple instances of an engine	One group per algorithm engine	Provides a hook for parallel partition processing
Offset checkpoint	Records replay and recovery position	Per-engine committed offsets and slot checkpoints	Supports deterministic replay and recovery logic
Retention policy	Preserves selected records for debugging and comparison	Short retention for transient states and longer retention for metrics	Supports offline analysis with controlled storage cost
Schema version	Protects interface compatibility	Mandatory version field in message headers	Prevents silent data–contract mismatch

**Table 7:** Representative Channel Fading and Path Loss Components.

Category	Type	Model/Component	Description
Large-scale fading	Basic path loss	FSPL or 3GPP-inspired loss	Scenario-dependent path loss under line-of-sight (LOS) or non-line-of-sight (NLOS) conditions.
Large-scale fading	Additional loss	Clutter, shadowing, atmospheric loss	Environment-dependent attenuation for dense urban, urban, suburban, rural, and satellite paths.
Small-scale fading	Flat fading	Rayleigh, Rician, Nakagami-*m*	Captures different LOS and NLOS fading severities.
Small-scale fading	Frequency-selective fading	Abstracted by effective rate mapping	Used when packet-level channel equalization is outside the emulation scope.

**Table 8:** Reference parameters for performance emulation.

Parameter	Value
Service area and reference load	100km×100km area with 600 reference users
Node inventory and locations	5 fixed macro BSs, 2 fixed UAV access nodes, and 4 LEO beam nodes modeled by ground-projected beam centers
Macro BS node configuration	35 m height, 46 dBm transmit power, 15 dBi antenna gain, maximum abstract access capacity of 64 users per slot
UAV access-node configuration	250 m height, 37 dBm transmit power, 10 dBi antenna gain, maximum abstract access capacity of 42 users per slot
LEO beam-node configuration	550 km altitude, 43 dBm transmit power, 32 dBi transmit gain, 3 dBi receive gain, maximum abstract beam capacity of 130 users per slot
Carrier frequencies	2 GHz macro, 2.5 GHz UAV, and 20 GHz satellite carriers
Bandwidth, noise figure, and access threshold	20 MHz per node, 7 dB noise figure, RSSI >−110 dBm, and $\gamma_{u,n}^{\mathrm{dB}}>-3$ dB; $\gamma_{u,n}^{\mathrm{dB}}$ is SNR in the reference emulator because cochannel interference is not modeled
Maximum candidate-link range	45 km terrestrial and 1600 km satellite
Clutter loss and shadowing	4 dB macro clutter loss, 2 dB UAV/satellite clutter loss, 6 dB terrestrial shadowing standard deviation, and 2.5 dB satellite shadowing standard deviation
Satellite excess loss	$2.6+0.002\;d_{u,n}^{\mathrm{km}}$ dB atmospheric loss and 0.2–2.8 dB random rain attenuation
Instantaneous service-delay abstraction	Node-dependent baselines of 4, 6, and 28 ms for macro BS, UAV, and satellite nodes, respectively, plus propagation delay and a random term of 1–6 ms for terrestrial links or 10–35 ms for satellite links; service delay adds $4d_{u}/\max\left(r_{u},0.05\right)$ ms; no inter-slot queue evolution
User distribution and service mix	55% uniform and 45% hotspot sampling; service-class probabilities of 45% eMBB, 20% URLLC, and 35% mMTC
Service demand and delay bound	Lognormal demand with nominal demand/delay-bound pairs of 10 Mbps/80 ms for eMBB, 4 Mbps/35 ms for URLLC, and 1.2 Mbps/200 ms for mMTC; log-domain standard deviation of 0.38 and demand floor of 0.2 Mbps
Access selection parameters	$\alpha_{R}^{\mathrm{LA}}=0.12$, $\beta_{\ell }^{\mathrm{LA}}=85$, and $\beta_{\ell }^{\mathrm{EN}}=0.35$
Swarm-search parameters	P=8, I=5, ω=0.55, $c_{1}=c_{2}=1.15$, $\Delta_{F}=0.04$, and reference $w^{\mathrm{SW}}=\left(0.28,0.23,0.17,0.17,0.10\right)$
Handover event parameters	$T_{\mathrm{dwell}}=3$ slots, $T_{\mathrm{pp}}=10$ slots, switching penalty 0.10, and reversal penalty 0.25 within two slots
Handover trace and sensitivity range	30-slot generated traces; mobility scale 0.6–2.2; dwell-duration sweep 1–7 slots
Scheduling parameters	$\delta_{d}=0.5$, $\rho_{U}=0.75$, reference $\theta_{\mathrm{alloc}}=\theta_{\mathrm{eval}}=0.85$, $\kappa_{C}=0.55$, $C_{\max}=13B_{\mathrm{MHz}}=260$ Mbps, $\lambda_{\tau }=55$, $\tau_{\min}=20$ ms, and $\rho_{\mathrm{res}}=0.5$
Workload, mobility, outage, and demand sweeps	Access workload from 200 to 1200 users, mobility scale factors 0.6–2.2, outage intensity 0–100%, and demand multipliers 0.6–1.6
Access, handover, routing, and scheduling policies	RSS-only, load-aware, and entropy-based access; threshold-only, dwell-time-aware, and swarm-guided handover; terrestrial-only, satellite-assisted, and adaptive routing; equal, PF-inspired demand-normalized, utility-weighted, and QoS-oriented heuristic scheduling
Random seed configuration	Base seed 2026 with seven deterministic replicas

## Data Availability

The original contributions presented in this study are included in the article. Further inquiries can be directed to the corresponding author.

## Abbreviations

The following abbreviations are used in this manuscript.

BS	Base station
eMBB	Enhanced mobile broadband
DVB	Digital Video Broadcasting
FSPL	Free-space path loss
HARQ	Hybrid automatic repeat request
ID	Identifier
IoT	Internet of Things
LEO	Low Earth orbit
MAC	Medium access control
mMTC	Massive machine-type communication
NR	New Radio
NTN	Non-terrestrial network
PHY	Physical layer
PF-inspired	Proportional-fairness-inspired scheduling
QoS	Quality of service
RSS	Received signal strength
RSSI	Received signal strength indicator
SINR	Signal-to-interference-plus-noise ratio
SNR	Signal-to-noise ratio
STIN	Satellite–terrestrial integrated network
UAV	Unmanned aerial vehicle
URLLC	Ultra-reliable low-latency communication
